# A Prevalência da Hipotensão Ortostática e a Distribuição da Variação Pressórica no Estudo Longitudinal da Saúde do Adulto

**DOI:** 10.36660/abc.20180354

**Published:** 2020-06-29

**Authors:** Ana Paula Costa Velten, Isabela Bensenor, Paulo Lotufo, José Geraldo Mill

**Affiliations:** 1 Universidade Federal do Espirito Santo VitóriaES Brasil Universidade Federal do Espirito Santo, Vitória, ES - Brasil; 2 Universidade de São Paulo São PauloSP Brasil Universidade de São Paulo, São Paulo, SP – Brasil

**Keywords:** Hipotensão Ortostática/epidemiologia, Prevalência, Doença da Artéria Coronariana, Pressão Arterial, Posição Ortostática

## Abstract

**Fundamento:**

A hipotensão ortostática (HO) tem sido negligenciada na clínica não havendo estudos sobre sua prevalência na população brasileira.

**Objetivo:**

Determinar a prevalência de HO e a variação da pressão arterial (PA) após manobra postural no Estudo Longitudinal da Saúde do Adulto.

**Métodos:**

No presente estudo descritivo da linha de base (N = 14.833 indivíduos, 35-74 anos), os participantes ficavam deitados por 20 minutos e então levantavam ativamente, com a medida da PA em supino e aos 2, 3, e 5 minutos de ortostase. A HO foi definida por queda ≥ 20 mmHg na PA sistólica e/ou queda ≥ 10 mmHg na PA diastólica aos 3 minutos, sendo determinada a sua prevalência com intervalo de confiança de 95% (IC_95%_). A distribuição da variação da PA após a manobra postural foi determinada numa subamostra (N = 8.011) após remoção de participantes com morbidade cardiovascular e/ou diabetes.

**Resultados:**

A prevalência de HO foi de 2,0% (IC_95%_: 1,8 – 2,3), crescente com a idade. Se o critério for a mesma queda pressórica em qualquer das medidas, a prevalência aumenta para 4,3% (IC_95%_: 4,0 – 4,7). Em presença de HO houve relato de sintomas (tontura, escotomas, náuseas, etc.) em 19,7% dos participantes (IC_95%_: 15,6 – 24,6) e em apenas 1,4% (IC_95%_: 1,2 – 1,6) dos sem HO. Os escores-Z −2 das variações da PA antes e após manobra postural na subamostra foram de −14,1 mmHg na PA sistólica e −5,4 mmHg na diastólica.

**Conclusão:**

A prevalência de HO varia em função do momento da aferição da PA. Os pontos de corte atuais podem subestimar a ocorrência de HO na população. (Arq Bras Cardiol. 2020; 114(6):1040-1048)

## Introdução

Estudos longitudinais têm mostrado que a hipotensão ortostática (HO) é preditor de aumento do risco de mortalidade geral e de outros agravos à saúde, como doença arterial coronariana e cerebrovascular, fibrilação atrial, insuficiência cardíaca e novos casos de hipertensão.^1 - 5^

Diretrizes atuais definem a HO como a redução sustentada de 20 mmHg da Pressão Arterial Sistólica (PAS) e/ou de 10 mmHg da Pressão Arterial Diastólica (PAD) dentro de 3 minutos após a adoção da ortostase.^6^ A mesma diretriz sugere uma redução de 30 mmHg na PAS em indivíduos hipertensos como o critério mais adequado.

Desde a primeira definição de HO,^7^ a quantidade de estudos investigando a prevalência desse achado na população geral tem sido pequena, havendo grande divergência na metodologia da medida da pressão arterial (PA) e até mesmo nos critérios de definição. A maior parte dos estudos tem sido realizada em populações específicas, como idosos, portadores de diabetes, hipertensão, doença de Alzheimer, indivíduos hospitalizados ou institucionalizados.^8^

Mesmo sendo uma avaliação simples e barata, a medida da PA em manobra postural não é usual na prática clínica e poucos estudos epidemiológicos avaliaram essa medida, seus fatores associados e implicações no estado geral de saúde. A prevalência de HO encontrada em populações mais próximas da geral varia muito em função da idade, indo, em sua maioria, desde cerca de 5% (45 a 64 anos) até 30% em estudos apenas com idosos (> 65 anos).^4 , 9^ Praticamente a totalidade dos estudos foi realizada em populações da América do Norte,^5 , 10^ Europa^1 , 2^ e Ásia,^11^ e nenhum deles levou em consideração o critério de queda de 30 mmHg na PAS para hipertensos.

O objetivo deste estudo foi determinar a prevalência de HO em uma população brasileira, verificar sua associação com sintomas e descrever a distribuição da variação da PA após a manobra postural.

## Métodos

### Desenho e população de estudo

**Figura 1:**
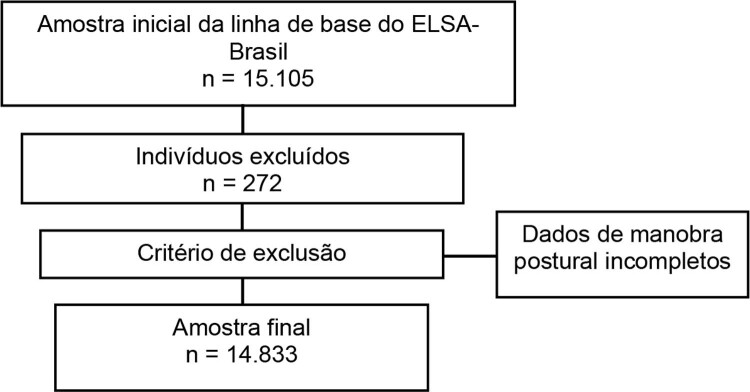
- *Fluxograma do estudo.*

Trata-se de um estudo descritivo realizado com dados coletados na linha de base (2008-2010) do Estudo Longitudinal de Saúde do Adulto (ELSA-Brasil), com uma coorte de 15.105 servidores públicos de ambos os sexos, de 35 a 74 anos de idade, cujo objetivo principal foi de determinar a incidência de doenças crônicas e os seus determinantes na população brasileira. A pesquisa vem sendo conduzida em seis centros de investigação sediados em instituições públicas de ensino superior e pesquisa, sendo os participantes os servidores públicos, ativos ou aposentados, destas instituições. Detalhes sobre a amostragem, recrutamento e dados coletados na linha de base foram publicados anteriormente.^12 , 13^ Neste estudo foram incluídos todos os participantes do ELSA-Brasil, exceto os que não possuíam dados completos da manobra postural. A amostra final foi composta por 14.833 indivíduos ( Figura 1 ).

### Manobra postural e hipotensão ortostática

Para a realização da manobra postural, o participante permanecia deitado por cerca de 20 minutos enquanto era submetido ao protocolo de aferição do índice tornozelo-braquial (ITB). Foram obtidas três medidas da PA no braço direito na posição supina com intervalo de dois minutos entre elas. Utilizou-se a média das duas últimas medidas como o valor da PA em supino. Em seguida, o aferidor instruía o participante a se levantar (se necessário com ajuda) e a manter postura ereta, com apoio apenas dos pés. A PA foi novamente medida aos 2, 3 e 5 minutos após adoção da ortostase, com o braço do participante sem apoio.^14^ Em formulário próprio, o aferidor deveria anotar a presença de sintomas relatados espontaneamente (tontura, alterações visuais, náusea, etc.). Em função da intensidade dos sintomas, o protocolo podia ser alterado fazendo-se as medidas da PA na posição sentada.

Houve uma rotina de treinamento, certificação e recertificação periódica dos aferidores. Supervisores treinados e certificados em nível central treinaram as equipes locais.^14^

Todas as medidas da PA foram obtidas com aparelho oscilométrico validado (Omron HEM 705CPINT, Japão)^15^ com tamanho do manguito escolhido de acordo com a circunferência do braço. Em 27 participantes foi necessária a utilização do esfigmomanômetro de mercúrio (Unitec, Brasil) por falha na leitura do aparelho oscilométrico. Outros 14 participantes não conseguiram manter a ortostase para todas as medidas pressóricas e tiveram incremento pressórico com o retorno à posição supina. Para estes fez-se uma correção baseada na média dos deltas pressóricos dos indivíduos que permaneceram eretos com o mesmo valor de queda pressórica.

Definiu-se HO pela presença de queda da PAS de ≥ 20 mmHg e/ou da PAD de ≥ 10 mmHg na medida de 3 minutos após adoção da ortostase.^6 , 7^ Posteriormente foi feita avaliação de prevalência considerando a queda pressórica em qualquer medida ou ainda adotando-se a queda de ≥ 30 mmHg na PAS dos hipertensos.

### Análise estatística

A prevalência de HO foi determinada por sexo, faixa etária, raça/cor e escolaridade. Os dados de prevalência foram apresentados como frequência e intervalo de confiança (IC) de 95%. A fim de afastar a influência da presença de doenças cardiovasculares ou diabetes, a prevalência de HO foi recalculada para uma subamostra gerada pela remoção dos indivíduos com hipertensão (em uso ou não de anti-hipertensivos), diabetes, auto-relato de insuficiência cardíaca, doença coronária prévia (infarto ou colocação de stent) e acidente vascular cerebral (AVC). Foram descritos ainda as médias e desvios padrões de idade de cada subamostra. Além disso foi verificada a prevalência de sintomas relacionados à mudança postural nos indivíduos com e sem HO.

Descreveu-se também a média e o desvio padrão (DP) da variação da PA (delta da pressão: PA em ortostase menos PA em supino) por faixa etária e em geral, tanto para a variação da PAS, quanto para a variação da PAD. Por fim foram calculadas as prevalências de HO considerando as quedas pressóricas aos 2, 3 e 5 minutos, assim como para o critério de queda de ≥ 30 mmHg na PAS aos 3 minutos, nos hipertensos. Ainda traçou-se um diagrama de Venn com as medidas dos três momentos. As análises foram efetuadas utilizando os softwares Microsoft Office Excel e o IBM SPSS Statistics 21.

### Questões éticas

O ELSA-BRASIL foi submetido e aprovado nos Comitês de Ética em Pesquisa das instituições envolvidas e todos os participantes assinaram o termo de consentimento.^16^

## Resultados

A prevalência de HO por sexo, faixa etária, raça/cor e escolaridade na população do estudo e na subamostra é apresentada na Tabela 1 . A média de idade na população total do estudo foi maior do que na subamostra (52,1 ± 9 anos *versus* 49,1 ± 8,2 anos, respectivamente; p < 0,01). As prevalências de HO nestes dois grupos foram, respectivamente, de 2,0% (IC_95%:_ 1,8 – 2,3) e 1,5% (IC_95%_: 1,3 – 1,8). Considerando em toda a população os indivíduos com menos de 60 anos e com 60 anos ou mais, a prevalência de HO foi de 1,6% (IC_95%_: 1,4 – 1,9) e 3,2% (IC_95%_: 2,8 – 4,1), respectivamente. Na subamostra, esses valores foram 1,4% (IC_95%_: 1,1 – 1,7; média de idade = 47,2 anos) e 2,6% (IC_95%_: 1,8 – 3,8; média de 64,3 anos), respectivamente. O efeito da idade fica mais claro agrupando-se os indivíduos por décadas. Observa-se que a prevalência abaixo de 55 anos é praticamente idêntica na população total e na subamostra. A partir desta idade, a subamostra apresenta prevalências menores. Outro fator com impacto na prevalência foi a escolaridade, havendo aumento progressivo da prevalência entre os participantes de menor escolaridade, tanto na população total como na subamostra.

**Tabela 1:** Prevalência de hipotensão ortostática segundo dados sociodemográficos, ELSA-Brasil (2008 – 2010)

Variáveis		Hipotensão ortostática		

		Presente	Total	Prevalência (IC 95%*)
**População do estudo (n = 14.833)**				

Sexo	Masculino	135	6.796	2,0 (1,7 – 2,4)
	Feminino	165	8.037	2,0 (1,8 – 2,4)
Faixa etária	35 a 44 anos	39	3.298	1,2 (0,9 – 1,6)
	45 a 54 anos	93	5.825	1,6 (1,3 – 2,0)
	55 a 64 anos	116	4.157	2,8 (2,3 – 3,3)
	65 a 74 anos	52	1.553	3,3 (2,6 – 4,3)
Cor ou raça	Preta	59	2.342	2,5 (2,0 – 3,2)
	Parda	81	4.110	1,9 (1,6 – 2,4)
	Branca	139	7.679	1,8 (1,5 – 2,1)
	Amarela/indígena	13	525	2,5 (1,5 – 4,2)
Escolaridade	Até fundamental completo	57	1.883	3,0 (2,3 – 3,9)
	Médio completo	110	5.133	2,1 (1,8 – 2,6)
	Superior completo	133	7.817	1,7 (1,4 – 2,0)
Total		300	14.833	2,0 (1,8 – 2,2)

**Subamostra da população do estudo† (n = 8.011)**				

Sexo	Masculino	56	3.289	1,7 (1,3 – 2,2)
	Feminino	66	4.722	1,4 (1,1 – 1,8)
Faixa etária	35 a 44 anos	33	2.570	1,3 (0,9 – 1,8)
	45 a 54 anos	50	3.388	1,5 (1,1 – 1,9)
	55 a 64 anos	30	1.688	1,8 (1,2 – 2,5)
	65 a 74 anos	9	365	2,5 (1,3 – 4,6)
Raça	Preta	16	1.223	1,3 (0,8 – 2,1)
	Parda	39	2.219	1,8 (1,3 – 2,4)
	Branca	63	4.181	1,5 (1,2 – 1,9)
	Amarela/indígena	3	284	1,1 (0,4 – 3,1)
Escolaridade	Até fundamental completo	15	645	2,3(1,4 – 3,8)
	Médio completo	39	2.643	1,5 (1,1 – 2,0)
	Superior completo	68	4.723	1,4 (1,1 – 1,9)
Total		122	8.011	1,5 (1,3 – 1,8)

*IC 95%: intervalo de confiança de 95%. †: População do estudo após a exclusão de hipertensos, usuários de anti-hipertensivos, diabéticos, com história de insuficiência cardíaca, doença coronária grave, infarto e acidente vascular cerebral.

Foram referidas alterações de protocolo em 775 (5,2%) indivíduos. Em 33,7% destes casos (260 indivíduos ou 1,8% da população total), relatou-se a ocorrência de sinais e sintomas sugestivos de HO (tontura, dificuldade em permanecer em pé sem apoio, náuseas, e mais raramente vômito). As mudanças de protocolos nos demais casos decorreram, em geral, de limitações físicas que dificultavam a realização da manobra, uso do braço ou perna esquerda (ITB), e do esfigmomanômetro de mercúrio.

O relato de sintomas associados à HO ocorreu em apenas 1,4% (IC_95%_: 1,2 – 1,6) dos indivíduos sem HO, valor este que subiu para 19,7% (IC_95%_: 15,6 – 24,6) naqueles com HO e para 43% (IC_95%_: 33,0 – 53,6) quando a HO era definida por queda de ambas as pressões.

Os valores médios e o DP das variações das PAS e PAD na manobra postural em toda a coorte e na subamostra estão descritas por sexo e faixa etária na Tabela 2 . Observa-se que, na média, as variações pressóricas são positivas, sem diferenças entre sexos e idade.

**Tabela 2:** Variação das pressões sistólica e diastólica (mmHg) aos 3 minutos, na população total do estudo e na subamostra da população do estudo, por sexo e faixa etária, ELSA-Brasil (2008 – 2010)

Faixa etária por sexo		Δ PAS (mmHg)				Δ PAD (mmHg)			

		Média (µ)	Desvio padrão (σ)	µ-2σ	µ-3σ	Média (µ)	Desvio padrão (σ)	µ-2σ	µ-3σ
**População do estudo (n = 14.833)**									

Total	Total	3,62	9,72	−15,81	−25,53	7,05	6,56	−6,07	−12,64
	Masculino	3,80	9,51	−15,21	−24,72	7,33	6,63	−5,93	−12,56
	Feminino	3,47	9,89	−16,31	−26,20	6,81	6,50	−6,18	−12,67
35 a 44 anos	Total	4,05	8,34	−12,63	−20,97	8,67	6,34	−4,00	−10,34
	Masculino	3,99	8,44	−12,89	−21,33	9,22	6,13	−3,05	−9,18
	Feminino	4,10	8,25	−12,40	−20,65	8,19	6,48	−4,76	−11,24
45 a 54 anos	Total	3,59	9,06	−14,53	−23,59	7,29	6,51	−5,74	−12,26
	Masculino	4,04	8,74	−13,44	−22,18	7,81	6,69	−5,57	−12,26
	Feminino	3,21	9,30	−15,39	−24,69	6,85	6,33	−5,82	−12,15
55 a 64 anos	Total	3,23	10,70	−18,16	−28,86	6,15	6,44	−6,74	−13,18
	Masculino	3,27	10,56	−17,85	−28,40	6,22	6,38	−6,55	−12,94
	Feminino	3,20	10,81	−18,41	−29,22	6,09	6,49	−6,89	−13,38
65 a 74 anos	Total	3,91	11,81	−19,70	−31,51	5,13	6,64	−8,15	−14,79
	Masculino	3,86	11,21	−18,57	−29,78	4,55	6,57	−8,58	−15,15
	Feminino	3,96	12,38	−20,79	−33,17	5,70	6,67	−7,63	−14,30

Subamostra da população do estudo* (n=8.011)									

Total	Total	3,77	8,93	−14,09	−23,03	7,48	6,43	−5,39	−14,96
	Masculino	3,76	8,83	−13,90	−22,73	7,89	6,40	−4,90	−15,79
	Feminino	3,78	9,01	−14,23	−23,24	7,19	6,44	−5,70	−14,38
35 a 44 anos	Total	4,17	8,35	−12,53	−20,88	8,70	6,30	−3,91	−17,39
	Masculino	3,99	8,53	−13,08	−21,61	9,25	6,16	−3,07	−18,49
	Feminino	4,32	8,21	−12,10	−20,31	8,27	6,38	−4,49	−16,54
45 a 54 anos	Total	3,59	8,67	−13,76	−22,43	7,31	6,35	−5,39	−14,62
	Masculino	3,92	8,33	−12,74	−21,07	7,83	6,28	−4,74	−15,66
	Feminino	3,36	8,89	−14,43	−23,32	6,95	6,37	−5,79	−13,90
55 a 64 anos	Total	3,50	9,80	−16,11	−25,91	6,37	6,38	−6,38	−12,74
	Masculino	3,00	9,75	−16,49	−26,23	6,41	6,41	−6,40	−12,82
	Feminino	3,79	9,83	−15,87	−25,70	6,34	6,36	−6,38	−12,69
65 a 74 anos	Total	3,89	10,80	−17,71	−28,52	5,64	6,88	−8,13	−11,27
	Masculino	3,65	10,92	−18,19	−29,11	4,80	6,62	−8,43	−9,61
	Feminino	4,08	10,73	−17,39	−28,12	6,29	7,04	−7,79	−12,57

Δ: diferença das pressões antes e após ortostase; µ: média; σ: desvio padrão; *: População do estudo após a exclusão de hipertensos, usuários de anti-hipertensivos, diabéticos, auto-relato de insuficiência cardíaca, doença coronária grave, infarto e acidente vascular cerebral.

**Figura 2:**
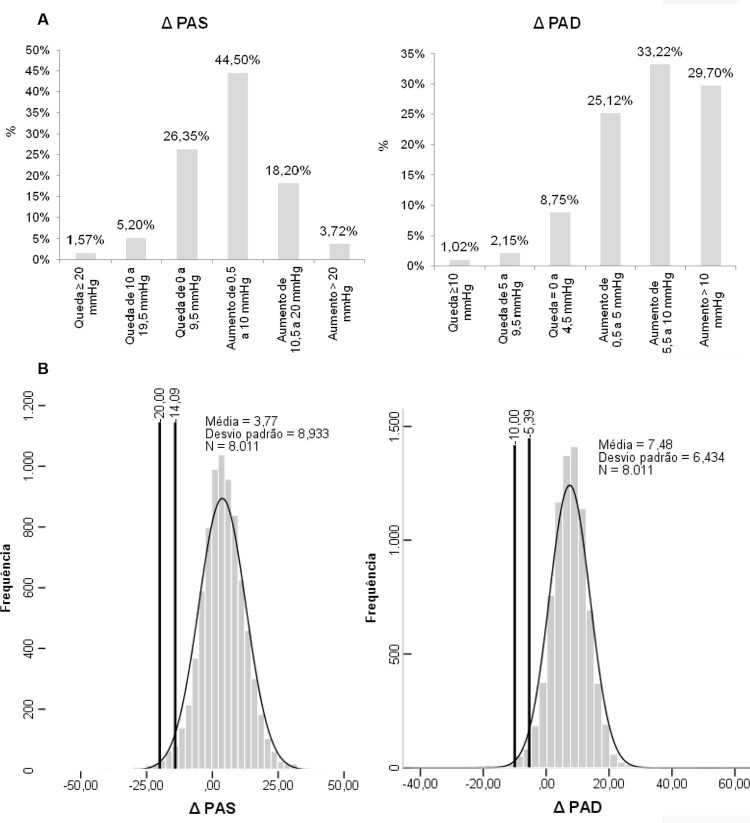
A) Alteração das pressões sistólica e diastólica após 3 minutos de ortostase por faixa de diferença em toda a população do estudo, ELSA-Brasil (2008 – 2010). B) Histograma dos deltas das pressões arteriais sistólica e diastólica aos 3 minutos após a ortostase, na subamostra da população do estudo, ELSA-Brasil (2008 – 2010).

A Figura 2A apresenta as variações pressóricas por faixa de diferença. Observa-se que geralmente essa oscilação situa-se de −10 a +10 mmHg na PAS e de aumentos de até 10 mmHg na PAD. Há aumento da PAS em 66,4% da população e em 88,0% da PAD. A Figura 2B contém o histograma das variações na subamostra. Estão sinalizados os valores da média menos dois DP e o atual valor de referência. As variações seguem distribuição normal e semelhante, e os pontos de corte atuais situam-se entre dois e três DP abaixo da média.

A prevalência aos 3 minutos considerando o critério de queda de 30 mmHg nos indivíduos hipertensos foi de 1,5% (IC_95%_: 0,3 – 1,7), totalizando 222 participantes. Ainda sobre a medida dos 3 minutos, considerando a queda em ambas as pressões, a prevalência situou-se em 0,6% (IC_95%_: 0,5 – 0,7), queda somente na PAS prevalência em 1,6% (IC_95%_: 1,4 – 1,8) e queda somente na PAD em 1,0% (IC_95%_: 0,9 – 1,2).

**Figura 3:**
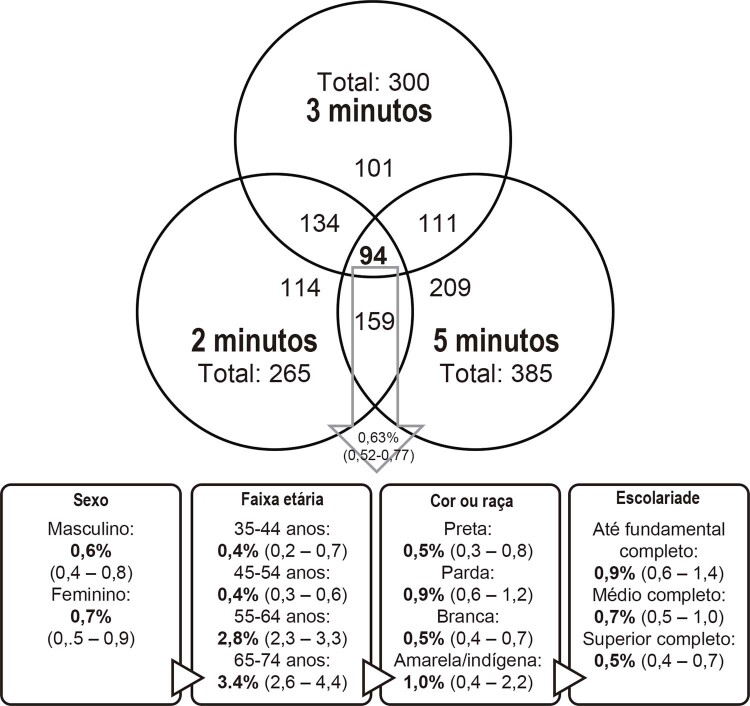
- *Diagrama de Venn sobre a hipotensão ortostática aos 2, 3 e 5 minutos, incluindo a descrição da prevalência total e segundo dados sóciodemográficos dos indivíduos com hipotensão ortostática nos três momentos, ELSA-Brasil (2008 – 2010).*

A Figura 3 apresenta o diagrama de Venn para a HO aos 2, 3 e 5 minutos. Nota-se que 265 indivíduos tiveram HO aos 2 minutos, prevalência de 1,8% (IC_95%:_ 1,6-2,0); e 385 indivíduos aos 5 minutos, 2,6% (IC_95%:_ 2,4-2,9). Em toda amostra, 94 indivíduos apresentaram HO em todas as medidas. Novamente não se observou diferença significativa entre sexos e raça/cor, mas houve uma progressão importante com a idade e com níveis mais baixos de escolaridade. A presença de sintomas relacionados à HO foi relatada em 10,2% (IC_95%:_ 7,1 – 14,4) dos que apresentaram HO aos 2 minutos e 17,4% (IC_95%_: 13,9 – 21,5) dos que apresentaram aos 5 minutos.

A prevalência de HO considerando a existência de queda pressórica aos 2 ou 3 minutos sobe para 2,9% (IC_95%:_ 2,7 – 3,2) e alcança 4,3% (IC_95%_: 4,0 – 4,7) quando considerada queda pressórica em pelo menos uma das três medidas. Na população acima de 60 anos esses valores seriam de 5,1% (IC_95%_: 4,4 – 5,9) e 7,3% (IC_95%_: 6,5 – 8,2), respectivamente.

## Discussão

Em nosso conhecimento, esse é o primeiro estudo de prevalência de HO em uma grande amostra da população brasileira. Destaca-se que o valor de 2,0% encontrado foi similar em homens e mulheres e apresentou nítido crescimento com a idade, notadamente a partir dos 55 anos. Na subamostra gerada com menor influência de fatores confundidores, a prevalência caiu para 1,5%. Essa queda decorreu principalmente das diferenças nas faixas superiores a 55 anos.

A comparabilidade de dados entre estudos sobre HO é difícil, dada a diversidade de características das populações, sobretudo no que diz respeito à faixa etária, e à heterogeneidade dos métodos utilizados na execução da manobra postural. Em populações não específicas, similares à geral, são encontradas prevalências que oscilam entre 2,73%^5^ a 58,6%.^17^ A mais baixa (2,73%) foi descrita nos participantes do Atherosclerosis Risk in Communities (ARIC) Study, cuja média de idade era de 53 anos, ou seja, similar à da linha de base do ELSA-Brasil. No ARIC a PA foi medida na posição supina e depois em pé a cada 30 segundos por 2 minutos, utilizando-se a média dessas medidas (exceto a primeira) para definir HO. Destaca-se que os participantes eram normotensos. Já para Cooke et al. (58,6%),^17^ a média de idade era de 73 anos e a PA foi medida de modo contínuo (batimento a batimento), durante 3 minutos em mesa de *tilt* com inclinação de 70^o^. A HO foi definida pela queda pressórica a qualquer momento durante o monitoramento, independente da duração. Assim, as diferenças de prevalência decorrem da diversidade de populações e de metodologia, tendo como traço comum apenas os pontos de corte da queda pressórica. Em nossa própria amostra considerando a queda pressórica de 2 ou de 3 minutos, ou a queda em qualquer medida, a prevalência cresce de 2,9% e 4,3%, respectivamente, sendo a última mais que o dobro da prevalência somente no terceiro minuto (2,0%) que tem sido o momento mais referido em estudos descritos na literatura.

Há grande variação em relação ao tempo da medida. Há estudos medindo a PA após 3 minutos da ortostase;^18^ após 1 e 3 minutos, considerando a queda em uma das duas medidas a partir da medida da posição supina;^19^ ou da medida com o participante sentado;^20^ medindo após 1 minuto;^3^ ou após 1, 2 e 3 minutos^21^ ou 1, 2 e 5 minutos,^22^ considerando a queda em qualquer medida; medindo de forma contínua, considerando a queda entre 60 e 110 segundos;^23^ existindo ainda outras variações.^11 , 24^

A diretriz atual recomenda a definição pela queda pressórica dentro de 3 minutos^6^ após a ortostase. Não há consenso, entretanto, sobre qual o melhor momento dentro deste período. A fim de determinar o tempo mais apropriado, um estudo^25^ avaliou 407 idosos (média de idade de 78,7 ± 7,8 nos com HO e 74,1 ± 8,6 nos sem HO aos 3 minutos) após 1, 3 e 5 minutos da ortostase. A prevalência encontrada foi de 21,86%, 21,37% e 19,90%, respectivamente, e os parâmetros associados à HO foram os mesmos nos três momentos. É destacado que 29 idosos apresentaram HO somente no primeiro minuto, 18 somente no terceiro e 12 somente no quinto. Os autores sugerem a adoção de 1 minuto para utilização na prática clínica por necessitar de menor tempo (o que é importante principalmente em idosos) e por identificar a maioria dos casos. Destaca-se que se a definição de HO fosse feita com base na queda pressórica a qualquer tempo, a prevalência seria maior.

Outros estudos^26 , 27^ sugerem avaliações mais prolongadas, de até 10 minutos, uma vez que muitos participantes desenvolveram HO tardiamente. Em nosso estudo, com média de idade mais baixa (52,1 ± 9,1 anos), alguns participantes também desenvolveram queda pressórica mais tardia, visto que a prevalência aos 3 minutos foi maior que aos 2, e que a de 5 minutos foi maior que ambas.

É preciso ter cautela na interpretação dos dados provenientes de monitorização contínua da PA após adoção de ortostase. Nesses casos, um declínio pressórico fisiológico pode ser esperado após adoção da ortostase, principalmente nos idosos, pela redução súbita do retorno venoso e do débito sistólico, até que os mecanismos de compensação estabilizem a PA. Finucane et al.,^10^ notou estabilização dentro de 30 segundos em indivíduos entre 50 a 59 anos, e com mais de 30 segundos nos indivíduos mais velhos. Tendo em vista essa queda inicial, considerar a queda independente do momento que ela ocorre como HO pode ser inapropriado. Os trabalhos que assim procedem têm encontrado prevalências muito altas, como 58,6%, encontrada por Cooke et al.,^17^ Tais valores devem conter grande quantidade de falsos positivos. Cooke et al.,^17^ mencionam que, caso fosse considerada a queda sustentada da PA com duração mínima de 60 segundos, a prevalência cairia para 23,3% e para apenas 9%, caso fosse considerada a manutenção por 180 segundos.

Após essas considerações sobre a heterogeneidade das populações e métodos, a comparação entre os trabalhos deve ser cautelosa. Estudos em populações com faixa etária semelhante à do ELSA apresentam prevalências entre 2,73%^5^ a 7,4%;^28^ com destaque para os artigos referentes às coortes ARIC^4 , 5 , 28^ e Malmo Preventive Project (MPP).^1 , 2^ As variações decorrem de exclusões nas amostras em função dos desfechos de cada artigo. A maioria dos artigos do ARIC apresenta prevalência de cerca de 5%. Em todos eles a média de idade foi de cerca de 53 anos. Os artigos do MPP apresentam prevalência de cerca de 6% e amostras bem semelhantes, com média de idade de 48 anos.

Destaca-se que estudos de prevalência em indivíduos com menos de 45 anos são escassos. Encontramos apenas um^20^ com indivíduos de 18 a 100 anos (média de 49 anos). Entretanto, a prevalência por faixa etária não foi mencionada.

O aumento da prevalência de HO com o envelhecimento estaria ligado a uma série de causas. Pode-se citar as alterações na função barorreflexa, respostas vasoconstritoras inadequadas, redução da complacência cardíaca e vascular, diminuição do volume sanguíneo e menor eficiência dos músculos esqueléticos de atuar como bomba facilitadora do retorno venoso.^29^ Além disso, em idades mais avançadas é maior a prevalência de hipertensão arterial, condição associada à HO. Isso, entretanto, parece não ter ocorrido em nosso estudo haja vista que o aumento pressórico na população total do ELSA foi similar àquele observado na subamostra, tanto em relação à pressão sistólica como diastólica ( Tabela 2 ).

Em nossa população, além da faixa etária, uma menor escolaridade também indicou tendência de aumento de HO, traço este observado tanto na amostra global como na subamostra, com uma atenuação neste último grupo. Ressalta-se que as diferenças de idade são um fator adjacente a estes achados, já que os grupos com menor escolaridade apresentaram média de idade maior (56,5 anos na categoria de menor escolaridade e de 51,9 na de maior, na população total; na subamostra essas médias foram de 53,4 anos e de 49,3 anos, respectivamente).

Com relação à presença de sintomas, observamos que a prevalência de HO é significantemente maior quando algum sintoma característico de queda no fluxo sanguíneo cerebral é relatado, principalmente quando a queda ocorre em ambas as pressões. No Cardiovascular Health Study,^18^ 20% dos indivíduos com HO apresentaram sintomas e no Rotterdam Study^30^ este indicador foi de 13,9%. Esses valores são próximos aos detectados no ELSA-Brasil, confirmando que a HO é assintomática na maioria dos indivíduos. A presença de sintomas é relevante para iniciar novas avaliações diagnósticas e tomar decisões terapêuticas. No entanto, não há diretrizes sobre a tomada de decisões clínicas nos portadores de HO, mas sem sintomas.^31^

A distribuição dos deltas das pressões resultaram em escores-Z de −2 a −14,09 mmHg para a PAS e de −5,39 mmHg na PAD na subamostra de pacientes sem a presença de hipertensão (medicados ou não), diabetes, histórico de insuficiência cardíaca, doença coronária grave, infarto ou AVC. Rose et al.,^5^ em amostra de 6.951 participantes, após a exclusão dos hipertensos, encontraram valor semelhante no percentil 5 da queda da PAS (−15,25 mmHg) mesmo utilizando um método diferente. Ressalta-se que os documentos que definiram a HO^6 , 7^ relatam o ponto de corte de −20 mmHg na PAS e −10 mmHg na PAD. Considerando que a variação pressórica apresenta distribuição gaussiana, os pontos de corte atualmente recomendados para definir presença de HO estariam além daqueles previstos por um critério estatístico padrão, isto é, considerar variação excessiva os indivíduos que se situam nos 5% inferiores de distribuição da curva. A definição de ponto de corte além deste limite aumenta a probabilidade de falsos negativos, ou seja, indivíduos portadores de HO que não receberiam este diagnóstico e a orientação adequada em função do estabelecimento de pontos de corte com base empírica e não com achados experimentais.

Com relação à prevalência de HO, considerando o critério de uma queda ≥ 30 mmHg em hipertensos, nota-se pequena diminuição da prevalência (de 2% para 1,5%), obviamente pelo deslocamento para a esquerda do ponto de corte. A sugestão de 30 mmHg é justificada na diretriz^6^ devido à maior PA inicial dos hipertensos. Entretanto, nos hipertensos do ELSA, menos da metade apresentava PA não controlada, ficando a dúvida em como proceder nessa situação, já que a mesma não é abordada na definição. Não encontramos alusão sobre a prevalência em hipertensos utilizando esse critério em outros estudos.

Sobre a prevalência nas demais medidas, destaca-se que houve aumento com o passar do tempo, e que muitos indivíduos apresentaram HO somente em uma das três medidas pressóricas. A prevalência simultânea nos três momentos foi de apenas 0,6%, tendo também relação com o envelhecimento, e 4,3% em qualquer um dos momentos. Uma análise associativa com os principais fatores relacionados à HO encontrados na literatura pode indicar qual(s) o(s) momento(s) mais adequado(s) para avaliação da HO nessa população.

Sobre a presença de sintomas e a prevalência de HO nos três momentos, os indivíduos que tiveram HO aos 3 minutos foram os que mais relataram sintomas. Vale lembrar que não há informação sobre o momento exato do relato, podendo o sintoma ter sido relatado/ocorrido logo após a ortostase ou mais próximo à medida pressórica de 5 minutos. A presença de sintomas, principalmente tontura e síncope, pode ter grande impacto na saúde dos indivíduos, já que pode afetar sua mobilidade e segurança.

A extensão dos achados da nossa amostra para a população geral deve ser feita com cautela por tratar-se de uma coorte profissional. Entretanto, a amostra é grande para permitir análises de subgrupos e grande parte do espectro de variação de idade, raça/cor e escolaridade existente na população brasileira está representada em ambos os sexos. Portanto, na ausência de dados populacionais, os dados do ELSA constituem atualmente a melhor referência para a presença de HO na população brasileira.

## Conclusão

A prevalência de HO em uma amostra de trabalhadores públicos brasileiros foi de cerca de 2% ao se levar em consideração as medidas pressóricas obtidas 3 minutos após adoção da ortostase. A prevalência é igual em ambos os sexos e a idade é o fator que mais influi na prevalência. A medida pressórica aos 3 minutos após adoção de ortostase é a que melhor se correlaciona com a presença de sintomas. Os atuais pontos de corte (-20 mmHg na PAS e -10 mmHg na PAD) podem subestimar a real ocorrência de HO na população.
