# Supplementary material for: A Prevalência da Hipotensão Ortostática e a Distribuição da Variação Pressórica no Estudo Longitudinal da Saúde do Adulto
Source: Arq Bras Cardiol. 2020 Jun 29;114(6):1040–8. [Article in Portuguese] doi: 10.36660/abc.20180354 (PMC8416122; doi:10.36660/abc.20180354)
Supplement: Translation

# Translation: Prevalence of Orthostatic Hypotension and the Distribution of Pressure Variation in the Longitudinal Study of Adult Health

**Authors:** Ana Paula Costa Velten, Isabela Bensenor, Paulo Lotufo, José Geraldo Mill

**Affiliations:** 1 Brazil Universidade Federal do São Mateus, Vitória, ES - Brazil; 2 Brazil Universidade de São Paulo, São Paulo, SP – Brazil

**Keywords:** Hypotension, Orthostatic/epidemiology, Prevalence, Coronary Artery Disease, Blood Pressure, Standing Position

## Abstract

**Background:**

Orthostatic hypotension (OH) has been neglected in clinical practice, and there are no studies on its prevalence in the Brazilian population.

**Objective:**

To determine the prevalence of OH and blood pressure (BP) changes after the postural change maneuver in participants of the Longitudinal Study of Adult Health.

**Methods:**

In this descriptive study of baseline data (N = 14,833 adults, ages 35 – 74 years), participants remained lying down for 20 minutes and subsequently stood up actively. BP measurements were taken while the participants were supine and at 2, 3, and 5 minutes after standing. OH was defined as a reduction of ≥ 20 mmHg in systolic BP and/or a reduction of ≥ 10 mmHg in diastolic BP at 3 minutes, and its prevalence was determined with a 95% confidence interval (CI). The distribution of BP variation after the postural change maneuver was determined in a subsample (N = 8,011) obtained by removing patients with cardiovascular morbidity and/or diabetes.

**Results:**

The prevalence of OH was 2.0% (95% CI: 1.8 – 2.3), increasing with age. If the criterion applied were a BP reduction during any measurement, the prevalence would increase to 4.3% (95% CI: 4.0 – 4.7). Symptoms (dizziness, scotoma, nausea, etc.) were reported by 19.7% of participants (95% CI: 15.6 – 24.6) with OH and 1.4% (95% CI: 1.2 – 1.6) of participants without OH. The −2 Z-scores of BP variation before and after the postural change maneuver in the subsample were −14.1 mmHg for systolic BP and −5.4 mmHg for diastolic BP.

**Conclusion:**

Prevalence of OH varies depending on when BP is measured. Current cutoff points may underestimate the actual occurrence of OH in the population. (Arq Bras Cardiol. 2020; 114(6):1040-1048)

## Introduction

Longitudinal studies have shown that orthostatic hypotension (OH) is a predictor of increased risk of overall mortality and other health issues, such as coronary artery disease, cerebrovascular disease, atrial fibrillation, heart failure, and new cases of hypertension.^1 - 5^

Current guidelines define OH as a sustained reduction of 20 mmHg in systolic blood pressure (SBP) and/or 10 mmHg in diastolic blood pressure (DBP) within 3 minutes after standing.^6^ The same consensus statement also suggests a reduction of 30 mmHg in SBP in individuals with hypertension as a more adequate criterion.

Since the first definition of OH,^7^ the number of studies investigating the prevalence of this finding in the general population has been low, and there has been great divergence in the methodology for measuring blood pressure (BP) and even in the criteria for definition. Most of the studies have been carried out in specific populations, such as elderly patients; patients with diabetes, hypertension, or Alzheimer’s disease; or hospitalized or institutionalized individuals.^8^

Even though it is a simple and inexpensive assessment, BP measurement with the postural change maneuver is not commonly used in clinical practice, and few epidemiological studies have evaluated the measurement, its associated factors, and its implications for general health status. The prevalence of OH found in populations that most approximate the general population differs greatly according to age, varying, mostly, from around 5% (45 to 64 years of age) to 30% in studies including only elderly individuals (> 65 years).^4 , 9^ Practically all of the studies carried out have been in populations from North America,^5 , 10^ Europe,^1 , 2^ and Asia,^11^ and none of them have applied the criteria of a reduction of 30 mmHg in SBP for patients with hypertension.

This study’s objectives were to determine the prevalence of OH in a Brazilian population, to verify its association with symptoms, and to describe the distribution of BP changes following the postural change maneuver.

## Methods

### Study design and population

This is a descriptive study carried out with data collected at the baseline (2008 – 2010) of the Longitudinal Study of Adult Health (ELSA-Brazil), with a cohort of 15,105 civil servants of both sexes, between 35 and 74 years of age, whose main objective was to determine the incidence of chronic diseases and their determinants in the Brazilian population. The study is being carried out in six centers of investigation located in public higher education and research institutes, the participants being active or retired civil servants from these institutes. Details on sampling, recruiting, and data collected at the baseline have been previously published.^12 , 13^ This study included all the participants of the ELSA-Brazil, with the exception of those who did not have complete data on the postural change maneuver. The final sample was composed of 14,833 individuals ( Figure 1 ).

**Figura 1:** Fluxograma do estudo.

### Postural change maneuver and orthostatic hypotension

To perform the postural change maneuver, participants remained lying down for approximately 20 minutes while they were submitted to the protocol for measuring ankle-brachial index (ABI). Three BP measurements were obtained in the right arm in the supine position, with two-minute intervals between them. The average of the last two measurements was used as the supine BP value. Subsequently, the assessor instructed the participant the stand up (with help, if necessary) and to maintain an upright posture, standing only on his or her feet. BP was measured again at 2, 3, and 5 minutes after standing, without supporting the participant’s arm.^14^ Assessors were instructed to take note of spontaneously reported symptoms (dizziness, visual alterations, nausea, etc.) on a specific form. Depending on the intensity of symptoms, it was possible to alter the protocol and measure BP in the seated position.

Assessors received routine training, certification, and periodic recertification. Supervisors who were trained and certified on the central level trained local teams.^14^

All BP measurements were obtained using a validated oscillometric device (Omron HEM 705CPINT, Japan),^15^ and cuff size was chosen according to arm circumference. It was necessary to use a mercury sphygmomanometer (Unitec, Brazil) for 27 participants, owing to failure to read the oscillometric device. Another 14 participants were unable to maintain orthostasis for all BP measurements, and their BP increased when they returned to the supine position. For these individuals, a correction is made based on the average BP variations or the individuals who remained standing with the same values of reduced pressure.

OH was defined as the presence of a reduction in SBP of ≥ 20 mmHg and/or DBP of ≥ 10 mmHg in the measurement at 3 minutes after standing.^6 , 7^ Subsequently, prevalence was evaluated considering a BP reduction in any measurement or applying a reduction of ≥ 30 mmHg in SBP for patients with hypertension.

### Statistical analysis

The prevalence of OH was determined by sex, age range, race/color, and level of schooling. Data on prevalence were shown as frequency and 95% confidence intervals (CI). With the aim of avoiding the influence of the cardiovascular diseases or diabetes, the prevalence of OH was recalculated for a subsample generated by the removal of patients with hypertension (whether or not they were using anti-hypertensive medication), diabetes, self-reported heart failure, prior coronary disease (infarction or stent placement), and stroke. The average and standard deviation (SD) for age were also described for each subsample. Furthermore, the prevalence of symptoms related to postural change in individuals with or without OH was verified.

Average and SD were also described for variations in BP (pressure variation: orthostatic BP minus supine BP) by age range and overall, for variations in both SBP and DBP. Finally, the prevalence of OH was calculated considering pressure reductions at 2, 3, and 5 minutes and applying the criterion of a reduction of ≥ 30 mmHg in SBP at 3 minutes for patients with hypertension. A Venn diagram was also developed for the three different measurements. Analyses were carried out using Microsoft Office Excel and IBM SPSS Statistics 21.

### Ethics

The ELSA-Brazil received approval from the Research Ethics Committees of the institutions involved, and all participants signed a consent form.^16^

## Results

The prevalence of OH by sex, age range, race/color, and level of schooling in the study population and the subsample is shown in Table 1 . The average age of the total study population was higher than that of the subsample (52.1 ± 9 years *versus* 49.1 ± 8.2 years, respectively; p < 0.01). The prevalence of OH in these two groups was 2.0% (95% CI: 1.8 –2.3) and 1.5% (95% CI: 1.3 – 1.8), respectively. In the entire population, considering individuals under the age of 60 and those 60 or over separately, the prevalence of OH was 1.6% (95% CI: 1.4 – 1.9) and 3.2% (95% CI: 2.8 – 4.1), respectively. In the subsample, these values were 1.4% (95% CI: 1.1 – 1.7; average age of 47.2 years) and 2.6% (95% CI: 1.8 – 3.8; average age of 64.3 years), respectively. The effect of age is ever clearer when grouping individuals by decades. It was observed that the prevalence below the age of 55 was practically identical in the total population and the subsample. After this age, the subsample presented a lower prevalence. Another factor that impacted prevalence was level of schooling; there was a progressive increase in prevalence among participants with lower levels, in both the total population and the subsample.

**Table 1:** Prevalence of orthostatic hypotension by sociodemographic data, ELSA-Brazil (2008 – 2010)

Variables		Orthostatic hypotension		

		Present	Total	Prevalence (95% CI*)
**Study population (n = 14,833)**				

Sex	Male	135	6,796	2.0 (1.7 - 2.4)
	Female	165	8,037	2.0 (1.8 - 2.4)
Age range	35 to 44 years	39	3,298	1.2 (0.9 - 1.6)
	45 to 54 years	93	5,825	1.6 (1.3 – 2.0)
	55 to 64 years	116	4,157	2.8 (2.3 - 3.3)
	65 to 74 years	52	1,553	3.3 (2.6 - 4.3)
Color/race	Black	59	2,342	2.5 (2.0 - 3.2)
	Mixed	81	4,110	1.9 (1.6 - 2.4)
	White	139	7,679	1.8 (1.5 - 2.1)
	Yellow/Indigenous	13	525	2.5 (1.5 - 4.2)
Highest level of schooling completed	Primary	57	1,883	3.0 (2.3 - 3.9)
	Secondary	110	5,133	2.1 (1.8 - 2.6)
	Tertiary	133	7,817	1.7 (1.4 - 2.0)
Total		300	14,833	2.0 (1.8 – 2.2)

**Subsample of the study population† (n = 8,011)**				

Sex	Male	56	3,289	1.7 (1.3 - 2.2)
	Female	66	4,722	1.4 (1.1 - 1.8)
Age range	35 to 44 years	33	2,570	1.3 (0.9 - 1.8)
	45 to 54 years	50	3,388	1.5 (1.1 - 1.9)
	55 to 64 years	30	1,688	1.8 (1.2 - 2.5)
	65 to 74 years	9	365	2.5 (1.3 - 4.6)
Color/race	Black	16	1,223	1.3 (0.8 - 2.1)
	Mixed	39	2,219	1.8 (1.3 - 2.4)
	White	63	4,181	1.5 (1.2 - 1.9)
	Yellow/Indigenous	3	284	1.1 (0.4 - 3.1)
Highest level of schooling completed	Primary	15	645	2.3(1.4 - 3.8)
	Secondary	39	2,643	1.5 (1.1 - 2.0)
	Tertiary	68	4,723	1.4 (1.1 - 1.9)
Total		122	8,011	1.5 (1.3 - 1.8)

*95% CI: 95% confidence interval; †: Study population following the exclusion of patients with hypertension, diabetes, history of heart failure, severe coronary disease, infarction, and stroke, as well as those using anti-hypertensive medication.

Protocol changes were reported in 775 (5.2%) individuals. Of these cases, 33.7% (260 individuals, 1.8% of the total population) reported the occurrence of signs and symptoms suggestive of OH (dizziness, difficulty standing without support, nausea, and rarely vomiting). Protocol changes in other cases generally resulted from physical limitations that complicated performance of the maneuver, use of the arm or the left leg (ABI), and use of the mercury sphygmomanometer.

Report of symptoms associated with OH occurred in only 1.4% (95% CI: 1.2 – 1.6) of individuals without OH; this value increased to 19.7% (95% CI: 15.6 –24.6) in individuals with OH and to 43% (95% CI: 33.0 – 53.6) when defining OH as a reduction in both pressures.

Average values and SD of SBP and DBP with the postural change maneuver for the entire cohort and the subsample are described by sex and age range in Table 2 . It was observed that, on average, pressure variations were positive, with no differences between sexes and age groups.

**Table 2:** Variation in systolic and diastolic pressure (mmHg) at 3, in the total study population and the subsample, by sex and age range, ELSA-Brazil (2008 – 2010)

Age range by sex		Δ SBP (mmHg)				Δ DBP (mmHg)			

		Average (µ)	Standard deviation (σ)	µ-2σ	µ-3σ	Average (µ)	Standard deviation (σ)	µ-2σ	µ-3σ
**Study population (n = 14,833)**									

Total	**Total**	3.62	9.72	−15.81	−25.53	7.05	6.56	−6.07	−12.64
	Male	3.80	9.51	−15.21	−24.72	7.33	6.63	−5.93	−12.56
	Female	3.47	9.89	−16.31	−26.20	6.81	6.50	−6.18	−12.67
35 to 44 years	Total	4.05	8.34	−12.63	−20.97	8.67	6.34	−4.00	−10.34
	Male	3.99	8.44	−12.89	−21.33	9.22	6.13	−3.05	−9.18
	Female	4.10	8.25	−12.40	−20.65	8.19	6.48	−4.76	−11.24
45 to 54 years	Total	3.59	9.06	−14.53	−23.59	7.29	6.51	−5.74	−12.26
	Male	4.04	8.74	−13.44	−22.18	7.81	6.69	−5.57	−12.26
	Female	3.21	9.30	−15.39	−24.69	6.85	6.33	−5.82	−12.15
55 to 64 years	Total	3.23	10.70	−18.16	−28.86	6.15	6.44	−6.74	−13.18
	Male	3.27	10.56	−17.85	−28.40	6.22	6.38	−6.55	−12.94
	Female	3.20	10.81	−18.41	−29.22	6.09	6.49	−6.89	−13.38
65 to 74 years	Total	3.91	11.81	−19.70	−31.51	5.13	6.64	−8.15	−14.79
	Male	3.86	11.21	−18.57	−29.78	4.55	6.57	−8.58	−15.15
	Female	3.96	12.38	−20.79	−33.17	5.70	6.67	−7.63	−14.30

Subsample of the study population* (n = 8,011)									

**Total**	**Total**	3.77	8.93	−14.09	−23.03	7.48	6.43	−5.39	−14.96
	Male	3.76	8.83	−13.90	−22.73	7.89	6.40	−4.90	−15.79
	Female	3.78	9.01	−14.23	−23.24	7.19	6.44	−5.70	−14.38
35 to 44 years	Total	4.17	8.35	−12.53	−20.88	8.70	6.30	−3.91	−17.39
	Male	3.99	8.53	−13.08	−21.61	9.25	6.16	−3.07	−18.49
	Female	4.32	8.21	−12.10	−20.31	8.27	6.38	−4.49	−16.54
45 to 54 years	Total	3.59	8.67	−13.76	−22.43	7.31	6.35	−5.39	−14.62
	Male	3.92	8.33	−12.74	−21.07	7.83	6.28	−4.74	−15.66
	Female	3.36	8.89	−14.43	−23.32	6.95	6.37	−5.79	−13.90
55 to 64 years	Total	3.50	9.80	−16.11	−25.91	6.37	6.38	−6.38	−12.74
	Male	3.00	9.75	−16.49	−26.23	6.41	6.41	−6.40	−12.82
	Female	3.79	9.83	−15.87	−25.70	6.34	6.36	−6.38	−12.69
65 to 74 years	Total	3.89	10.80	−17.71	−28.52	5.64	6.88	−8.13	−11.27
	Male	3.65	10.92	−18.19	−29.11	4.80	6.62	−8.43	−9.61
	Female	4.08	10.73	−17.39	−28.12	6.29	7.04	−7.79	−12.57

Δ: difference in pressure before and after standing; µ: average; σ: standard deviation; *: Study population following the exclusion of patients with hypertension, diabetes, history of heart failure, severe coronary disease, infarction, and stroke, as well as those using anti-hypertensive medication.

Figure 2A shows pressure variations by range of difference. It was observed that the variation was generally situated from −10 to +10 mmHg for SBP, with increases of up to 10 mmHg in DBP. SBP increased in 66.4% of the population, and DBP increased in 88.0%. Figure 2B contains the histogram of the variations in the subsample. Average values minus two SD and the current reference value are indicated. The variations follow normal and similar distribution, and the current cutoff points are located between two and three SD below the average.

**Figure 2:** – *A) Alteration in systolic blood pressure (SBP) and diastolic blood pressure (DBP) at 3 minutes after standing, by age group, in the entire study population, ELSA-Brazil (2008 – 2010). B) Histogram of SBP and DBP variation at 3 minutes after standing, in the subsample of the study population, ELSA-Brazil (2008 – 2010).*

The prevalence at 3 minutes, when applying the criterion of a reduction of 30 mmHg for individuals with hypertension was 1.5% (95% CI: 0.3 – 1.7), with a total of 222 participants. Furthermore, with respect to measurement at 3 minutes, considering a reduction in both pressures, prevalence was 0.6% (95% CI: 0.5 – 0.7); considering a reduction in SBP alone, prevalence was 1.6% (95% CI: 1.4 – 1.8), and, considering a reduction in DBP alone, it was 1.0% (95% CI: 0.9 – 1.2).

Figure 3 shows the Venn diagram for OH at 2, 3, and 5 minutes. It may be observed that 265 individuals presented OH at 2 minutes, a prevalence of 1.8% (95% CI: 1.6 – 2.0), whereas 385 individuals presented OH at 5 minutes, a prevalence of 2.6% (95% CI: 2.4 – 2.9). In the total sample, 94 individuals presented OH at all measurements. Once again, no significant differences were observed for sex or race/color, but there was an important progression with age and with lower levels of schooling. The presence of symptoms related to OH was reported in 10.2% (95% CI: 7.1 – 14.4) of individuals who presented OH at 2 minutes and 17.4% (95% CI: 13.9 – 21.5) of those who presented OH at 5 minutes.

**Figure 3:** Venn diagram for orthostatic hypotension at 2, 3, and 5 minutes, including description of total prevalence and prevalence by sociodemographic data of individuals with orthostatic hypotension during all three measurements, ELSA-Brazil (2008 – 2010).

The prevalence of OH, considering the existence of a reduction in pressure at 2 or 3 minutes, increases to 2.9% (95% CI: 2.7 – 3.2), and it reaches 4.3% (95% CI: 4.0 – 4.7) when considering a reduction in pressure in at least one of the three measurements. In the population over the age of 60, these values would be 5.1% (95% CI: 4.4 – 5.9) and 7.3% (95% CI: 6.5 – 8.2), respectively.

## Discussion

To the best of our knowledge, this is the first study on the prevalence of OH in a large sample of the Brazilian population. It is noteworthy that the finding of 2.0% was similar in men and women, and it showed clear growth with age, especially after the age of 55. In the subsample generated with fewer confounding factors, prevalence decreased to 1.5%. This decreased results mainly from differences in age ranges over 55.

The comparability of the data between studies on OH is complicated, given the diversity of the characteristics of the populations, especially with respect to age range, and to the heterogeneity of methods used to perform the postural change maneuver. In non-specific populations similar to the general population, prevalence is found to vary from 2.73%^5^ to 58.6%.^17^ The lowest (2.73%) was described in the participants of the Atherosclerosis Risk in Communities (ARIC) Study, whose average age was 53 years, making it similar to the baseline of the ELSA-Brazil. In the ARIC, BP was measured in the supine position and then while standing, every 30 seconds for 2 minutes, using the average of these measurements (excluding the first one) to define OH. It is worth noting that the participants were normotensive. In contrast, in Cooke et al. (58.6%),^17^ average age was 73 years, and BP was measured continuously (beat-to-beat), for 3 minutes on a tilt table at 70^o^. OH was defined as any drop in pressure at any moment during monitoring, regardless of duration. Thus, the differences in prevalence result from the diversity of the populations and the methods, the only common feature being the cutoff points for reduction in pressure. In our sample, considering a reduction in pressure at 2 or 3 minutes, or a reduction in any measurement, the prevalence increased to 2.9% and 4.3%, respectively; the latter is more than double the prevalence at the third minute alone (2.0%), which has been the most reported moment in studies described in the literature.

There is a great deal of variation with respect to timing of measurement. There are studies measuring BP after standing for 3 minutes;^18^ after 1 and 3 minutes, considering a reduction in one of the measurements in relation to the measurement in the supine position;^19^ with seated participants;^20^ after 1 minute;^3^ after 1, 2, and 3 minutes;^21^ after 1, 2, and 5 minutes;^22^ considering a reduction in any measurement; measuring continuously, considering a reduction between 60 and 110 seconds;^23^ and others.^11 , 24^

The current guidelines recommend defining OH as a reduction in pressure within 3 minutes^6^ after standing. There is, however, no consensus regarding the best moment within this period. In order to determine the most appropriate moment, one study^25^ evaluated 407 elderly patients (average age of 78.7 ± 7.8 for patients with OH and 74.1 ± 8.6 for patients without OH at 3 minutes) 1, 3, and 5 minutes after standing. The prevalences were 21.86%, 21.37%, and 19.90%, respectively, and the parameters associated with OH were the same during the three moments. It stands out that 29 elderly patients presented OH only during the first minute, 18 only during the third, and 12 only during the fifth. The authors suggest adopting 1 minute for use in clinical practice, because it requires less time (which is especially important in elderly patients) and identifies the majority of cases. It is worth noting that, were the definition of OH based on a reduction in pressure at any moment, the prevalence would be higher.

Other studies^26 , 27^ suggest longer evaluations, of up to 10 minutes, given that many participants develop OH in a delayed manner. In our study, with a lower average age (52.1 ± 9.1 years), some participants also developed more delayed reductions in pressure, given that prevalence was higher at 3 minutes than that at 2, and it was highest at 5.

It is necessary to use caution in interpreting data from continuous BP monitoring after standing. In these cases, a physiological decline in pressure may be expected after standing, especially in elderly patients, who are more susceptible to a sudden decrease in venous return and systolic output, until compensation mechanisms stabilize BP. Finucane et al.^10^ observed stabilization within 30 seconds in individuals between 50 and 59 years of age and within over 30 seconds in older individuals. Keeping this initial reduction in mind, it may be inappropriate to consider a reduction at any moment as OH. Studies conducted in this manner have found very high prevalences, such as that of 58.6% found by Cooke et al.^17^ These values must contain a large quantity of false positives. Cooke et al.^17^ mention that, were they to consider sustained reductions in BP with a minimum duration of 60 seconds, the prevalence would drop to 23.3%, and it would be only 9% if they considered reductions sustained for 180 seconds.

With these considerations regarding the heterogeneity of populations and methods, the comparison between studies should proceed cautiously. Studies in populations with age ranges similar to the ELSA-Brazil showed prevalences between 2.73%^5^ and 7.4%^28^ with the articles referring to to cohorts of the ARIC^4 , 5 , 28^ and the Malmo Preventive Project (MPP) standing out.^1 , 2^ The variations results from exclusions in the samples depending on the outcomes of each article. Most articles of the ARIC present a prevalence of approximately 5%. In all of them, the average age was around 53 years. The articles of the MPP present a prevalence of approximately 6% and very similar samples, with an average age of 48 years.

It is noteworthy that studies on prevalence in individuals under the age of 45 are scarce. We found only one^20^ with individuals from 18 to 100 years of age (average age of 49). However, the prevalence by age range was not mentioned.

The increase in the prevalence of OH with aging is linked to a series of causes. The following may be cited: changes in baroreflex function, inadequate vasoconstrictor responses, reduced cardiac and vascular compliance, decreased blood volume, and lower efficiency of skeletal muscles to act as a pump facilitating venous return.^29^ Moreover, as age advances, the prevalence of arterial hypertension is greater, and this condition is associated with OH. This, however, does not appear to have occurred in our study, seeing that the pressure increase in the total population for in the ELSA-Brazil was similar to that observed in the subsample, in relation to both SBP and DBP ( Table 2 ).

In our population, in addition to age range, lower level of schooling also indicated a tendency to increase OH; this feature was observed both in the general sample and the subsample, with an attenuation in the latter group. It stands out that these differences in age are factors adjacent to these findings, given that groups with lower levels of schooling presented a higher average age (56.5 years in the category with lower schooling and 51.9 in the total population; in the subsample, these averages were 53.4 and 49.3 years, respectively).

In relation to the presence of symptoms, we observed that the prevalence of OH was significantly higher when a symptom characteristic of a reduction in cerebral blood flow was reported, especially when the reduction occurred in both pressures. In the Cardiovascular Health Study,^18^ 20% of individuals with OH presented symptoms, and, in the Rotterdam Study,^30^ this indicator was 13.9%. These values are close to those detected in the ELSA-Brazil, confirming that OH is asymptomatic in most individuals. The presence of symptoms is relevant to indicate new diagnostic evaluations and make therapeutic decisions. In the meanwhile, there are no guidelines regarding clinical decision making in patients with OH who have no symptoms.^31^

The distribution of pressures variation resulted in Z scores from −2 to −14.09 mmHg for SBP and −5.39 mmHg for DBP in the subsample of patients without hypertension (with or without medication), diabetes, history of heart failure, severe coronary disease, infarction, or stroke. Rose et al.,^5^ with a sample of 6,951 participants, following the exclusion of patients with hypertension, found a value similar in the fifth percentile of the reduction in SBP (−15.25 mmHg), even using a different method. It is noteworthy that documents that defined OH^6 , 7^ reported a cutoff point of −20 mmHg for SBP and −10 mmHg for DBP. Considering that the pressure variation presents a Gaussian distribution, the cutoff points currently recommended for defining the presence of OH would go beyond those predicted by a standard statistical criterion, namely, considering excessive variation as individuals located in the 5% below the distribution of the curve. The definition of a cutoff point beyond this limit increases the probability of false negatives, or be it, individuals with OH who would not receive adequate diagnosis and advice depending on the establishment of cutoff points based on empirical and not experimental findings.

In relation to the prevalence of OH, considering the criterion of a reduction of ≥30 mmHg in patients with hypertension, a small decrease in prevalence was observed (from 2% to 1.5%), obviously owing to the fact that the cutoff point was shifted to the left. The suggestion of 30 mmHg is justified in the guidelines^6^ due to the higher initial BP in patients with hypertension. However, in patients with hypertension in the ELSA, fewer than half presented uncontrolled BP, posing a doubt as to how to proceed in this situation, given that the definition does not address it. We found no allusion to the prevalence of OH in patients with hypertension applying this criterion in other studies.

Regarding the prevalence in other measurements, it stands out that there was an increase as time progressed, and many individuals presented OH in only one of the three pressure measurements. The simultaneous present during the three moments was only 0.6%, also showing a relation to aging, and it was 4.3% in any measurement. An associative analysis with the main factors related to OH found in the literature may indicate which moment(s) would be most appropriate for evaluating OH in this population.

Regarding the presence of symptoms and the prevalence of OH during the three moments, the individuals who presented OH at 3 minutes were the ones who most reported symptoms. It is worth remembering that there is no information regarding the exact moment when the symptoms were reported, and the symptom may have been related or occurred immediately after standing or closer to the pressure measurement at 5 minutes. The presence of symptoms, especially dizziness and syncope, may have a great impact on the individuals’ health, given that it may affect their mobility and safety.

It is necessary to use caution when extending the findings in our sample to the general population, as it is a professional cohort. However, the sample was large enough to allow for subgroup analysis, and a great part of the spectrum of diversity in age, race/color, and level of schooling that exists in Brazil is represented in both sexes of the sample. Therefore, in the absence of population data, the data from the ELSA-Brazil currently constitute the best reference for the presence of OH in the Brazilian population.

## Conclusion

The prevalence of OH in a sample of Brazilian civil servants was around 2%, considering the pressure measurements obtained at 3 minutes after standing. This prevalence was equal in both sexes, and age was the factor that most influenced prevalence. The pressure measurement at 3 minutes after standing is the one that best correlates with the presence of symptoms. Current cutoff points (−20 mmHg in SBP and −10 mmHg in DBP) may underestimate the real occurrence of OH in the population.
